# Qualitative evaluation of the implementation and national roll-out of the NHS App in England

**DOI:** 10.1186/s12916-024-03842-w

**Published:** 2025-01-21

**Authors:** Claire Reidy, Chrysanthi Papoutsi, Sukriti KC, Bernard Gudgin, Anthony A. Laverty, Felix Greaves, John Powell

**Affiliations:** 1 Nuffield Department of Primary Care Health Sciences, University of Oxford, Radcliffe Observatory Quarter, Woodstock Road, Oxford, OX2 6GG UK; 2 School of Public Health, Imperial College London, London, UK; 3 Patient and Public Involvement Representative, Nuffield Department of Primary Care Health Sciences and member of the Advanced Research Computing Board, University of Oxford, Oxford, UK

**Keywords:** Patient portals, NHS App, Digital health, General practice, COVID Pass

## Abstract

**Background:**

The NHS App launched in 2019 as the ‘digital front door’ to the National Health Service in England with core features including General Practitioner (GP) appointment booking, repeat prescriptions, patient access to records and, later on, COVID-19 vaccination certification. Similar patient portals have been adopted in different formats and with variable levels of success. In this longitudinal study (2021–2023) we examined how the NHS App became implemented in the pandemic context and beyond.

**Methods:**

We recruited 88 participants in 62 qualitative interviews and four focus groups. Participants included patients, carers, members of the public, clinical/non-clinical NHS staff from five GP practices (where we also conducted over 60 h of observations) across England, as well as other industry, policy and civil rights stakeholders. Document analysis also contributed to participant recruitment and data interpretation. Data collection and analysis was informed by the Non-Adoption, Abandonment, Scale-up, Spread and Sustainability (NASSS) framework.

**Results:**

Our study identified the various ways in which complexity manifested as part of the implementation, use and roll-out of the NHS App. Patients had diverse (positive and negative) user experiences as the app evolved, with some of its features described as more useful than others (e.g. prescription ordering, COVID Pass). As the app primarily provided a gateway to general practice systems and infrastructures, not all features were available by default or consistently to all users, with information often appearing fragmented or system-facing (e.g. coded). NHS staff viewed the app as constituting core NHS infrastructure in the long term which made it appealing, even though initially there was less recognition of its immediate value. There was variable organisational capacity to enable implementation and to put in place processes and staff roles required to support patient adoption. Shifting emphasis towards in-person care, challenges with digital inclusion and controversies related to features such as patient access to own records further complicated roll-out.

**Conclusions:**

As the NHS App remains a complex innovation in a shifting landscape, it is clear ongoing work is needed to ensure its potential can be sustained to meet patient, service and policy needs.

**Clinical study registration:**

ISRCTN72729780.

**Supplementary Information:**

The online version contains supplementary material available at 10.1186/s12916-024-03842-w.

## Background

It is often suggested that digital health can offer opportunities and solutions [1] to improve access and efficiency and with the growing demand for access to primary care in England means services are looking for new ways to manage their capacity to support patients. In January 2019, the National Health Service (NHS) in England first introduced a new smartphone app for patients called the ‘NHS App’ (also accessible via a website). Originally designed to be the digital ‘front door’ to the health service in England, the app was rolled out nationally and was introduced to improve access to primary care services for all, enhance patient experience, save time in General Practitioner (GP) practices and promote self-care [2]. The intention was to ‘ease pressures on GPs’, ‘provide more effective, personalised care’ while ‘freeing up valuable clinician time’ [3] and the app. However, the literature on technology adoption and diffusion of innovation illustrates that the process of implementation is difficult and complex, with interacting influences from the features and useability of the technology itself, how the public and staff interact with and perceive it, the organisational culture and systems, and wider influences (e.g. the political and cultural context) [4].

The roll-out of the NHS App was in line with a general drive towards scaling up digital health in England [5]. The 2019 NHS Long Term Plan set out the ambition for patients to have access to their own health records and to be offered digital consultations with their GP [2], while NHS England’s ‘digital first’ strategy aimed to direct patients away from in-person engagements, through telephone, online or video consulting before face-to-face consultations (although policy-makers later reverted back to an increased emphasis on in-person consultations). The app was launched with core functions including prescription ordering, access to test results and own health records, General Practice (GP) appointment booking, organ donation preference setting, and display of the user’s NHS number and GP surgery details [6]. It is also linked to a symptom checker and the NHS 111 service (non-emergency medical helpline), and over time integrated with further services, such as the COVID-19 Pass (also known as a COVID vaccination status certification) [7], online consultations and personal health record platforms.

There was a rapid rollout plan aiming for full national usability by July 2019 [8] and access to care plans for those with long-term conditions by 2020 [9]. This timeline proved unrealistic, however, as the purpose and position of the app had to be re-negotiated in the context of other apps from the industry offering some similar features, and also due to the COVID-19 pandemic. The pandemic saw the introduction of ‘total triage’ in general practice [10], and the development of the NHS COVID-19 app (a separate track and trace product) became a priority. The NHS App still remained at the forefront of the policy agenda with new features and integrations added over time (such as vaccination certificates, linked profiles, GP messaging and authentication infrastructure), underpinned by extensive user research [11, 12]. As of December 2023, there were over 33 million downloads of the app [13].

There is widespread recognition that introducing new technologies in healthcare poses challenges and risks, but also offers opportunities [1]. The COVID-19 pandemic provided fertile ground for wider adoption of digital health innovations such as synchronous or asynchronous patient-clinician communication, patient portals and remote monitoring systems [14, 15]. This qualitative evaluation is part of wider work [16] providing the only national evaluation of a major component of the central plan to digitally transform the NHS in England. This evaluation enables an in-depth exploration of a digital health innovation which has been made available to all NHS users in a complex and multifaceted national health service, with consideration and examination of the wider context and competing and interacting local, regional, and national contexts.

## Research questions

This evaluation is the first to present how the NHS App has been used and experienced over time by patients, carers, members of the public, healthcare staff, delivery teams, digital health industry and other stakeholders, and whether and how the app has met varying needs in accessing and organising healthcare. More specifically we asked:

1. How did multiple interacting influences shape NHS App use, implementation and roll-out in general practice (and beyond)?
2. How did patients, carers and healthcare staff experience the integration of the NHS App in the health service? What were the perceived implications and transferable learnings for access, efficiency and safety for this and other digital health technologies in general practice?

## Methods

### Design

We used a longitudinal design with data generated through a number of qualitative methods (interviews, focus groups, observation and document analysis). We worked across different settings to explore technology implementation, roll-out and transferability and were able to extend our learning by comparing and contrasting between general practice sites. Longitudinal data collection (over 2 years between June 2021 and April 2023) facilitated the investigation of the NHS App as an evolving technology in a fast-changing policy and public health environment and exploration of sociotechnical processes over time (e.g. administrative processes, professional roles, norms around primary care access, and patient expectations).

#### Setting

We worked with five GP practices across England and used a staged approach to site identification and recruitment to achieve maximum variation in characteristics such as geographical location, ethnicity mix, patient list size, deprivation levels and digital maturity status (see Table 1). Sites were identified via Local Clinical Research Networks (LCRNs) and professional networks.

**Table 1:** Case site characteristics

	**Case site 1**	**Case site 2**	**Case site 3**	**Case site 4**	**Case site 5**
Location	South East England	East Midlands	North West England	North England	South East England
Urban/rural	Urban	Rural	Urban	Urban	Urban
Population served^a^	Mixed ethnic population: 3.5% mixed, 13.6% Asian, 4.5% black, 1.3% other non-white ethnic groups	Mostly white British: 1.3% non-white ethnic groups	Majority south Asian: 1.5% mixed, 66.9% Asian, 1.8% other non-white ethnic groups	Mixed ethnic population: 6.7% mixed, 20.2% Asian, 17.9% black, 1.9% other non-white ethnic groups	Mixed ethnic population: 7.3% mixed, 10.9% Asian, 20.1% black, 10.9% other non-white ethnic groups
Practice patient population size^b^	Large: 18,998	Average: 13,098	Average: 10,847	Small: 7385	Small: 3032
Deprivation level^c^	7	4	2	3	2
IT system used	EMIS	SystmOne	EMIS	SystmOne	EMIS
Digital maturity^d^	Level 4 (learning and improving)	Level 2: Traditional with lone innovator (ad hoc, demonstration)	Level 2: Traditional with lone innovator (ad hoc, demonstration)	Level 3: Digitally curious (experimenting)	Level 2: Traditional with lone innovator (ad hoc, demonstration)

^a^Estimated proportion of non-white ethnic groups in the practice population (weighted average over the contributing LSOAs)—Data source: Census 2011(Ethnicity by LSOA); HSCIC: numbers of Patients Registered at a GP Practice—April 2015 (Patients by LSOA) [17]

^b^9544 England average [17]

^c^Deprivation score (IMD 2019) of the area, 1 = most deprived, 10 = least deprived [17]

^d^Assessed using the Greenhalgh et al. digital maturity scale, from Level 1: Traditional (reactive) to Level 5: System-oriented (extending and spreading) [18]

Digital maturity was assessed using the Greenhalgh et al. digital maturity five-point scale [18], which considers the organisation’s readiness to plan and deliver a digital service (including measures to address digital inequalities), its capability (based on what level of digital measures are already present and running), and the organisational infrastructure (the underpinning material, regulatory, and human resource frameworks) that are in place to support further development of digital services.

The functionality offered through the NHS App to patients differed by practice (and over time). These are summarised in see Table 2 for clarification on the functionality offered to patients in each site.

**Table 2:** Functionality offered to patients through the NHS App according to each case site^a^

All Practices
All users were able to access their NHS number and GP surgery details, organ donation preference setting, and were able to link to a symptom checker and the NHS 111 service. These features did not require practices to enable access
All practices provided access to patients’ own health records and test results when patients requested this access or already had their own health record access digitally enabled through their practice previously. Practices had different protocols as to how access was given, and whether records would be assessed by staff to have data redacted.
All practices offered proxy access (available from July 2021) which was assessed on a case-by-case basis and access provided according to local protocols, and whether records would be assessed by staff to have data redacted. All practices provided access to the COVID Pass from May 2021
Functions available per Case site
Case site 1: a digital triage and online consultation service (additional add-on to the NHS App), online prescriptions (most prescriptions were encouraged to come through to this practice electronically, through the NHS App, another app or through the practice website). Initially provided appointment booking early on, but soon disabled this function, with no intention to reinstate
Case site 2: add-on personal health record functionality through a commercial provider (which allows access to secondary care health records and messaging, including photo messaging and questionnaires related to long term conditions^b^), and online prescriptions (although most prescriptions to the practice came through telephone)
Case site 3: online prescriptions. Initially used appointment booking early on, but disabled this function, with no intention to reinstate
Case site 4: a digital triage and online consultation service, online prescriptions (most prescriptions were encouraged to come through to this practice electronically, through the NHS App, another app or through the practice website)
Case site 5: online prescriptions (but patients were encouraged to continue using another app for this), appointment booking never used, but there were intentions to use this for nurse appointments in the future

^a^At the time of data collection

^b^This was commissioned at a local level by the local Clinical Commissioning Group (CCG)

### Data collection

We recruited 88 participants in 62 semi-structured and think-aloud interviews and four focus groups (see Table 3). Participants included patients, carers and members of the public (who used the app to different degrees or not at all), clinical and non-clinical NHS staff, and other stakeholders from the digital health industry, policy and commissioning, other public bodies, and civil rights organisations. Some of the participants discussed their experiences with the NHS App in a dual capacity, in terms of professional involvement but also personal use.

**Table 3:** Summary of data sources

• Interviews:
◦ 62 interviews with 66 participants comprising
▪ Semi-structured interviews (some involving more than one participant) with:
• 23 patients, carers and members of the public
• 23 healthcare staff (practice managers, GPs, nurses, healthcare assistants, admin and finance)
• 16 stakeholders (from commissioning, NHS agencies, policy, data privacy organisations, public bodies, industry)
▪ Think-aloud interviews with 4 patients
• Four condition-specific focus groups with 22 patients/carers living with diabetes, Long COVID, Parkinson’s, HIV
• Observations in five GP surgeries (60 h)
• Field notes (notes, photos, videos, screenshots)
• Document analysis of approximately 100 documents (policy blogs, GP documents/forms, government reports, social media discussions)

Semi-structured interviews explored individual experiences, while condition-specific focus groups (conducted with patients and carers only) allowed participants to collectively debate their views and perceptions. We followed a flexible topic guide adapted for different participant groups to gain an understanding of experiences with and perceptions of the app, how people used it, as well as background design and development processes, and the role the app played in organising care in general practice. Think-aloud interviews with four patients enabled direct in-depth observation of interaction with the app (e.g. viewing or using features on the app), facilitating articulation of how tasks were accomplished and challenges encountered. We captured these interactions in field notes, photos (of the participant’s use of the NHS App), screenshots, and videos, with participant permission. Interviews and focus groups took place on Microsoft Teams, over telephone or face-to-face at different stages of the pandemic and lasted between 16 and 72 min (average = 41 min) and 59 and 87 min (average = 73 min), respectively. All interviews and focus groups were digitally recorded and transcribed verbatim for analysis (with written or digitally recorded consent from participants).

The lead author (CR) conducted focused observations (face-to-face, totalling ~ 60 h) within each GP surgery, with extensive field notes and photos undertaken, to better understand the back-end operational and technical processes required to integrate the app in the service (e.g. managing digital appointment booking or prescription ordering). Observations facilitated the assessment of digital maturity within each practice site, the context of the practice, collection of NHS App-related and digital health promotion documents in GP surgeries and supported participant recruitment.

We collected a range of documents (e.g. policy blogs, Twitter discussions, government reports and plans, newspaper articles, and documents in GP surgeries—e.g. forms for patient digital health access) which helped inform recruitment but also contributed to data interpretation and context setting.

#### Participant recruitment and sampling

Patients and carers were primarily identified through theoretical and maximum variation sampling within case sites, through GP practice screening and selection or through invitation through practice patient groups (2–6 patients per site). Participant exclusion criteria included those who did not use NHS services. As data collection progressed, patients and carers were selected based on views that were either missing, or views that we wanted to explore in more depth, for example, patients who had attempted to gain proxy access, or patients who had supported a member of their family to access the app. Some patient and public participants were also directly recruited through voluntary and community organisations (such as groups representing different health conditions) or social media (Facebook groups and Twitter), e.g. those using the #NHSApp hashtag or using condition-specific groups.

Interviews and focus groups involved people living with a range of health conditions (see Table 4), including those living with, or carers of people living with, conditions such as HIV, long COVID (and related comorbidities), type 1 and type 2 diabetes, Parkinson’s, colitis, epilepsy, arthritis, skin conditions, cancer, asthma, amputations, high blood pressure, high cholesterol, fibromyalgia, Crohn’s disease, immune-compromised, kidney failure, allergies, severe intolerances, sight loss, mental health conditions and those having investigations for yet undetermined conditions (e.g. spinal stroke, eye conditions).

**Table 4:** Participant characteristics

Patient participants (*n* = 49)		
**Age (years): mean (SD), range**	53 (13.26), 20–79	
**Gender *n* (%)**	Female	30 (61%)
	Male	19 (39%)
**Ethnicity**^**a**^***n*(%)**	White British	33 (67%)
	Indian or British Indian	5 (10%)
	Pakistani or British Pakistani	2 (4%)
	Black British	3 (6%)
	Black Caribbean	2 (4%)
	Black African	1 (2%)
	British Bengali	1 (2%)
	White European	1 (2%)
	Turkish	1 (2%)
**Location in England *n* (%)**	South East	19 (39%)
	North West	11 (22%)
	East Midlands	8 (16%)
	North East	6 (12%)
	South West	4 (8%)
	West Midlands	1 (2%)
**Caring responsibilities *n* (%)**	Yes	11 (22%)
	No	38 (78%)
**NHS staff (*n* = 23)**		
**Roles *n* (%)**	Admin role	8 (35%)
	Practice Manager	5 (22%)
	GP	4 (17%)
	GP Partner	4 (17%)
	Nurse	1 (4%)
	Healthcare Assistant	1 (4%)
**Gender *n* (%)**	Female	17 (74%)
	Male	6 (26%)
**Ethnicity**^**a**^***n*(%)**	White British	9 (39%)
	Indian or British Indian	8 (35%)
	Pakistani or British Pakistani	2 (9%)
	Black Caribbean	1 (4%)
	Other Arabic	1 (4%)
	White European	1 (4%)
	Filipino	1 (4%)
**Location *n* (%)**	North West	7 (30%)
	South East	7 (30%)
	North East	6 (26%)
	East Midlands	3 (13%)
**Stakeholders (*n* = 16)**		
**Stakeholder number**	**Organisation**	**Role**
SH1	CCG^b^	Project Lead
SH2	CCG	Management
SH3	NHS App development and implementation teams	User Researcher
SH4	CCG	Management
SH5	CCG	Department Head
SH6	Data privacy organisation	Coordinator
SH7	NHS App development and implementation team	Department Head
SH8	Industry	Management
SH9	Industry	Director
SH10	Health tech support company	Implementation role
SH11	NHS App development and implementation teams	Director
SH12	CCG	Management
SH13	CCG	Implementation role
SH14	CCG	Implementation role
SH15	Professional Body	Stakeholder
SH16	Industry	CEO
**Organisations *n* (%)**	CCG	7 (44%)
	NHS App development and implementation teams	3 (19%)
	Industry	3 (19%)
	Data privacy organisation	1 (6%)
	Professional body	1 (6%)
	Health tech support company	1 (6%)
**Roles *n* (%)**	Management	4 (25%)
	Implementation role	3 (19%)
	Director	2 (13%)
	Department Head	2 (13%)
	CEO	1 (6%)
	Project Lead	1 (6%)
	User Researcher	1 (6%)
	Coordinator	1 (6%)
	Policy Officer	1 (6%)
**Gender *n* (%)**	Female	12 (75%)
	Male	4 (25%)
**Ethnicity**^**a**^***n*(%)**	White British	15 (94%)
	Indian or British Indian	1 (6%)
**Location *n* (%)**	South East	9 (56%)
	North West	5 (31%)
	East Midlands	2 (13%)

^a^Ethnicity data self-reported from participants

^b^Clinical Commissioning Groups—clinically-led statutory NHS bodies responsible for the planning and commissioning of health care services for their local area, although dissolved in July 2022 with their duties taken on by integrated care systems (ICS)

We used purposive sampling to recruit NHS staff (selecting participants from different professional roles to ensure a varied sample). NHS staff participants were identified and invited to participate through the case sites as staff working within the site. Exclusion criteria for NHS staff at selected GP sites included those who were not involved in any aspect of integration, roll-out or engagement with the NHS App.

Policy, commissioning, industry and other stakeholders were recruited using purposive and snowball sampling, taking into account relevant expertise in their professional role and involvement in the introduction and roll-out of the NHS App. Exclusion criteria included those not involved in any aspect of either the strategic planning, development, roll-out, policy making, communication or commissioning of the NHS App, or other relevant bodies and agencies (e.g. governance and data security). We contacted participants across a range of relevant organisations, drawing on authors’ professional networks and publicly available information.

Exclusion criteria for all participants included those patients, staff or stakeholders who might be unwilling or unable to give informed consent for participation.

### Data analysis

#### Theoretical framework

**Fig. 1:**
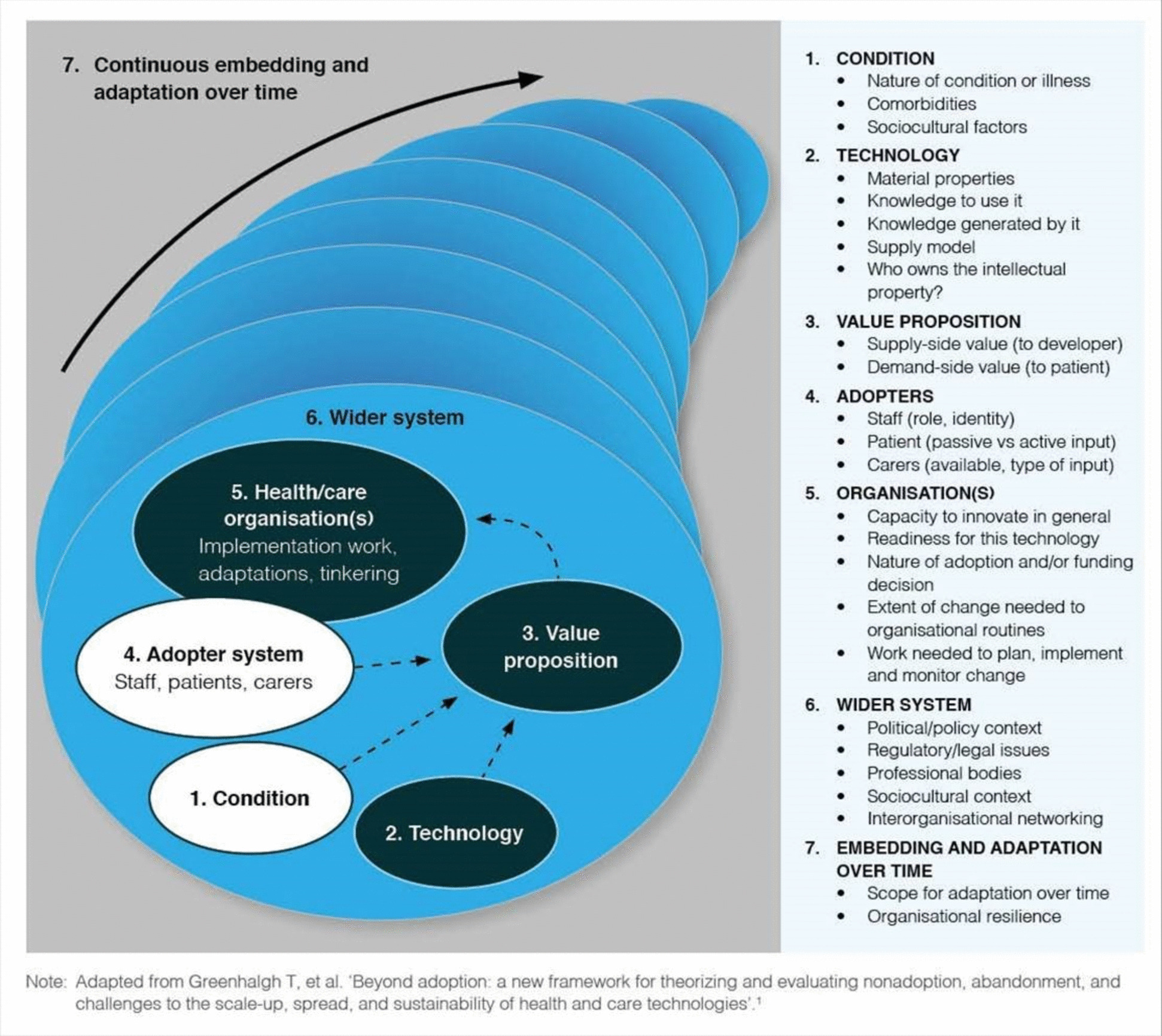
Non-adoption, abandonment and challenges to scale-up, spread and sustainability (NASSS) framework [19]

Data collection and analysis was informed by NASSS (non-adoption, abandonment, scale-up, spread, sustainability) (Fig. 1) an evidence-based framework developed to guide thinking on the implementation, roll-out and routine use of technology in healthcare [19]. By drawing on NASSS we have taken a complexity-informed approach [20] and explored how multiple interacting influences shaped NHS App use, implementation and roll-out in general practice, including differences in engagement with the NHS App depending on clinical needs and service configuration, the complexity of the technology itself and modifications to it over time, the value generated for different stakeholders, the role of NHS staff in its introduction, the underlying organisational structures in place and the complexity of wider NHS service provision.

#### Analysis

Analysis took place in parallel to data collection enabling us to refocus our methods over time, to account for a range of experiences and emergent themes until we were able to build a sufficient understanding [21]. It enabled us to consider changes as the app was rolled out, who we needed to speak to and what data we would need to capture to build on our evaluation of the NHS App roll-out, e.g. the development of the COVID Pass. Braun and Clarke’s six-step framework guided our thematic analysis, which began with data familiarisation, generation and iterative refinement of initial ideas and codes from transcripts and fieldnotes [22]. This was followed by inductive and deductive development of broader themes within and across NASSS domains, including comparing and contrasting similarities, differences, and connections in the data (especially across sites). Combining an inductive and deductive approach allowed us to pay attention to emergent as well as anticipated themes and to identify cross-cutting areas of complexity. We used NVivo 12 for data management.

### Patient and Public Involvement (PPI) contribution

Our Patient and Public Involvement (PPI) group was involved in the process of data analysis and sense-making through six workshops held at project initiation and set-up, through recruitment, and as data emerged. The workshops enabled a space for sharing, discussing and challenging insights gained, and to consider and prioritise approaches and directions for continuing data collection. Further, weekly team meetings with the PPI lead facilitated continued engagement throughout the study.

### Ethics

Ethical approval for this study was granted by the National Research Ethics Service and Health Research Authority (Reference 21/WS/0031).

## Results

Drawing on the NASSS framework, our analysis identified the various ways in which complexity manifested as part of the implementation, use and roll-out of the NHS App, including how multiple interacting influences shaped its trajectory. Illustrative quotes are provided under each NASSS domain.

### Domain 1: clinical condition

Participants with health conditions described different care pathways for the management of their health and shared different ways of engaging with the NHS App. For example, those with conditions primarily managed in general practice could access more functionality through the app (following up on investigations and test results from within the GP practice, and letters between GP practice and secondary care). Many of those primarily receiving care in hospital or community settings (e.g. patients living with Parkinson’s, HIV or type 1 diabetes) found the app supported their self-care less, as they were unable to access the majority of their test results or health records (unless their practice included a personal health record function add-on in the app through a commercial provider). This led some patients to express frustration with the app which they saw as reflecting system fragmentation and lack of joined-up care:

I’ve got now a multidisciplinary team behind me … [so] using those different authorities in the NHS, my medical records are not too complete…if I look at my [GP] medical record, I would be very surprised if it even says I’ve got Parkinson’s because I don’t interact with my GP surgery at all with Parkinson’s… I think [the NHS App] is great to have, [but] it’s gotta be able to interface with all the other potential medical people I use. (R5, Parkinson’s Focus Group, 57, Male, White British)

While the NHS App was designed to be used by anyone registered for NHS care (aged 13 or over), some patients and staff posited that it would be less relevant for those not managing long-term conditions (e.g. it was suggested young people were more likely to belong to this category) as they may not need to use its features, or the health service, as regularly:

Yeah I use like a health app on my phone like for my steps…I think my dad told me about it [the NHS App]… I’ll do it later…I just don’t feel like I need it at the minute… I don’t wanna do it… [it’s] for everyone and also for patients that might want to track anything on there…it can be for me, I just, I don’t know…I just don’t feel like I would use it very often that’s why… (P12, Site 3, 20, Female, British Indian)

### Domain 2: the technology

#### The app and its features, over time and in different settings

Compared to alternative patient portals already in use by GP practices, the NHS App required a more elaborate registration and authentication process (e.g. filling in an online or, usually, a physical form, plus the requirement to provide identity documents (see image 1, appendix 1), take a photo or video, facial recognition, and for the practice to enable a digital “linkage key”.) which in many cases made the app appear complex and difficult to navigate for patient users.

Apart from core functionality provided directly through the app, the rest of its features relied on background systems and infrastructures (e.g. GP IT systems, access to the Spine [23], Electronic Prescription Service data, Summary Care Record [24]) with the NHS App only functioning as gateway. This increased complexity as not all features were available by default and consistently to all users. For example, GP practices made decisions independently on whether they would enable appointment booking (including what types of appointments and how these would be displayed) through the app, which resulted in significant variability in whether, when and how this feature became available to patients:

…nobody understands how complicated booking practice nurse appointments are… and so we would never let them [patients] do that [online booking]… Because they would get it wrong… it depends on the nurses skill set if she’s the right nurse and how long it needs depends on certain things …there’s well over a 100 different types of [Practice Nurse] appointments… if [patients] speak to our staff they can signpost you… (S17, Site 4, Practice Manager)

Appointment booking was in fact disabled by most surgeries given triage protocols during the pandemic and demand pressures following consecutive lockdown periods (see image 2, appendix 1). There were some accounts of patients being able to use appointment booking on the app, especially before the pandemic, although this was not the case for most people we interviewed (who reported they would benefit from such a feature):

pre-Covid you had to put 75% of appointments available online for booking…Now the trouble with that is that goes against all sign posting practice… we spend our life trying to triage … to get things to the right clinician in the right time frame…[booking] straight into appointments online goes completely against all of that…While booking an appointment that they don’t need, you know, for a hay fever medication which they should just go and get from the chemist. There’s no way of filtering that. (S1, Site 1, Practice Manager)

Own health record access was controlled by local protocols and procedures which in some cases differed (at least in terms of their interpretation) between, and sometimes even within, GP practices (especially in relation to limiting access due to General Data Protection Regulation (GDPR) concerns):

there’s certain things in your records where it says… you don’t have access to this or you don’t have permission so I’ve actually gone into the surgery three or four times and said I want access to this part and quite often they say oh we don’t know how to do that… and for some reason my husband he’s got access to more sections on his than I have. (R6, Long covid Focus group, 48, Female, White British)

Even in cases where patients were provided with access to their own records, sometimes these were in a coded format, rather than designed for sharing with patients. Therefore, some patients found them to be lacking in meaning and relevance,

not that I can understand this [coded entry on the app]. (P24, Site 4, 62, Female, British Indian)

Over time the app became integrated with external commercial platforms, such as a personal health record platform, a digital triage and online consultation service (where patients submitted symptoms or requests to their GP practice), and other communication solutions. Again, these options were only available in GP practices or secondary care services using such external providers through local contracts and arrangements (e.g. independently or through their CCG. This increased variability in the features on the app, with additional options available for some patients and practices, but not others. It also complicated our interviews with patients as it was often unclear to us (and to them) what features were available to each of them, and at what point.

#### Knowledge required to access and use the app

Our data indicated a certain level of knowledge was needed to be able to request access to the app in the first place and make good use of available features. Yet, awareness of resources and guidance available to support the use of the app varied among staff and patients. Some suggested that the app should facilitate access in a more patient-facing way that does not assume advanced health literacy to interpret e.g. normal ranges for blood test results (something that was addressed in a latter version of the app):

…having an app like this does assume a level of health literacy that I don’t think the general population had, I mean again even somebody who has some medical awareness there’s some bits of it that I kind of look at and I go “what does that mean?” … [so] what is a blood test what is a normal range just having some access to some of those sort of information. (R2, Long covid Focus Group, 42, Female, White British)

In interviews and focus groups, there was confusion over what the NHS App was in the first place. Some patients thought it was the same as the COVID track and trace app used for contact tracing at the time. There was a general lack of awareness of what features (other than NHS COVID Pass) were available on the app and so patients and staff often articulated that more publicity and promotion was required.

Complexity emerged not just because practices had different processes and protocols in terms of enabling or disabling specific features on the app. Staff also needed to know how to interpret and activate these protocols (e.g. enabling proxy access or access to own records) so that features of the app would become usable for patients.

### Domain 3: the value proposition

#### Should this be the only app that practices use or one of many?

By designating the NHS App as the digital ‘front door’ to the NHS, policymakers aimed to promote access to services and support self-management, enabling patients to ‘be more equal partners’ in managing their health care. Members of the NHS App development teams saw it as a way to address inefficiencies, generate better (and less) use of appointments, and reduce the burden on services, such as through patients ordering prescriptions, monitoring test results or checking on referral progress. It was publicly pronounced as a ‘thin’ platform to which other services and third-party suppliers would connect, perhaps due to push-back from the industry about the NHS App’s potential to “stifle the market”:

Yes but if you’ve seen the App you will see that, so we got so much push back from third party suppliers about the NHS trying to stifle the market and Matthew [Gould]’s blog was very much the NHS App would remain very thin but nobody ever really kind of said what that meant… I think he meant it would be a platform that other services would connect to etc. … people would choose whatever, whatever kind of user interfacing they preferred. (SH7, Development team 2, Communications and stakeholder engagement)

However, the strategy or concept of a ‘thin’ app was not clearly characterised or explained to practices. Over time, and with the integration of popular features such as the COVID pass, the value was articulated in different ways to meet different needs and interests, given broader policy intentions and targets for widespread use.

#### Demand-side value: ‘Empowerment’ and ‘control’ for patients, reduced workload for practices?

For most patient participants value is often derived from relatively simple features, such as ordering prescriptions on the app (compared to calling or visiting the surgery). Security features, such as fingerprint scanning and face id to access the app were considered appealing to many users we spoke with and were especially important for those with health diagnoses they did not want shared outside the health service:

I’m really worried about my HIV status coming out but I would rather have my results on my phone, I don’t pass my phone to anybody so nobody can go onto it, you can’t go onto the App without your fingerprint so they can’t just see that App and think oh I’ve have a look and see what that is because they can’t get on it. (R4, HIV Focus Group, 47, Female, White British)

Having further features such as security codes for accessing the app sent through text messages provided reassurance (image 1, appendix 1).

When patients could access their own (or where they had proxy access rights to others’) health records, they reported an increased sense of control, for example, knowing what information was held about them, what consultations had occurred when, and what record was kept regarding those consultations. Some patients also saw such access to records as providing the ability to hold the service to account:

Well I suppose the purpose of the App should be for me to be able to know what information is kept about me, you know, with regards to whatever interaction I’ve had with the NHS… [if] the medical institution has any information about me, you know, I want to know what that information is. (P16, 56, Male, Black British)

However, given the availability of different features varied between settings, what patients expected as an added value from the app often did not materialise. This included quick and easy access to booking appointments through the app, viewing own health records or providing a history of encounters across the health service, rather than just in general practice.

With promised features not always realised, some patients were reluctant to shift to using the NHS App as they compared its value to other apps or portals with similar features they had already been using (e.g. pharmacy apps). They suggested added value would be generated if the NHS App could provide more interactive features, such as pop-up notifications when prescriptions were ready to collect, or when new test results, letters or referrals were issued. However, when pop-up notifications were enabled in newer versions of the app, technical issues meant some notifications appeared without any content.

From the perspective of general practice staff, the app did not always appear to have a clear and sustained value proposition in terms of supporting their practice, workload or engagement with patients, apart from the prescription ordering feature. They did not always perceive the app as having substantial clinical need or utility (at the time), for example, not tracking referrals, messaging patients, or other ways for the app benefiting patients directly, although general practice staff recognised it held significant potential in the future (and in some cases awareness about current app features was limited).

With the announcement of the COVID pass being added to the NHS App in April 2021, the value proposition became quickly associated with this feature and resulted in a rapid increase in downloads as well as increased awareness by practice staff. In addition, many patients that we spoke to had only heard about the NHS App in terms of the COVID pass, and some patient participants described being unsure what value the NHS App would hold for them when they were no longer required to use the COVID pass.

### Domain 4: the adopter system

#### Tensions between top-down implementation and staff capacity in general practice

At first, the introduction of the NHS App and its basic functions did not seem to require significant effort by GP staff, Yet, over time it became apparent that patients saw their surgery as the first point of contact e.g. how to register on the app, use it and request access to different features (such as proxy access, personal health records), also correcting personal details for travel purposes using the COVID pass. This meant surgery staff needed to become increasingly familiar with the app (and its various iterations) from a user and IT infrastructure perspective so they could answer such queries. Staff perceived a lack of available technical support, and some proposed the development of a toolkit to resolve issues, such as app error codes, or a central team or helpline to support with troubleshooting. Without a clear value proposition in terms of addressing workflow and demand pressures, and with little training or support to take on new roles and responsibilities related to the NHS App, some staff, especially practice managers, expressed frustration that the onus had been placed on them in a top-down way without meaningful consultation:

my staff haven’t had any help, haven’t had any training in how to support people [with the NHS App], most of them only know about it because they set it up for themselves… Or because they’re interested in NHS stuff and they do it out of the goodness of their hearts so they can help patients…And giving themselves a tutorial. (S17, Site 4, Practice Manager)

Staff also wanted to ensure the NHS App did not signal a complete move away from face-to-face engagement and digital approaches were used in the context of already established, trusted relationships with patients. This was particularly relevant for specific patient groups (e.g. those with language barriers or from particular cultural backgrounds) and those who needed more support to navigate the system through face-to-face contact rather than through complex online tools:

I think a lot of us are quite pro integrating technology into our systems it’s just integrating it in a way that doesn’t advantage the people that are already good at getting access to healthcare…So [digital triage tool]s are great, I would say a lot of the time they are used by people that already receive good healthcare… [unless] English isn’t your first language and you don’t have family support around you. (S14, Site 4, GP Partner)

Some functions (e.g. health record access) engendered more complexities than others, including fears that patients may be affected negatively by viewing their records in a system-facing rather than patient-facing format, leading to misunderstandings, additional appointments, as well as safeguarding concerns.

#### Variability in patient and carer experiences depending on features available

Patients, especially those living with long-term conditions, used the app to navigate the service and support self-care. In interviews, they discussed ordering prescriptions, checking symptoms, viewing test results, and (where available) booking appointments. However, many patients described app usage as occasional rather than regular, as opposed to banking, shopping or neighbourhood apps. Users with access to additional features (e.g. being able to view appointments and secondary care letters) appeared to have an enhanced experience of the app, as they perceived their care as more joined up (see images 4 and 5, appendix 1).

Those with proxy access on the NHS App (e.g. for family members) described being able to view health records more easily and conveniently “on the go”, and ordering prescriptions simply, which supported managing other responsibilities (including employment and caring for others). Yet the process of acquiring access was depicted as long and complex requiring persistence (taking more than 6 months or resulting in failure), as not all GP practices were familiar with protocols and technical processes (e.g. which boxes to select in IT systems) or carers were not always able to procure necessary identification documents. Proxy rights were also an issue when access requirements changed (e.g. COVID Pass age restriction).

#### Disparities around equitable use and access

While the NHS App was deemed by some to be more relevant to those living with and managing health conditions, some older adults who were managing (potentially multiple) health needs, or those with some specific cultural needs, described not being able to easily use the NHS App, or the NHS App not being something they wanted to use. Personal social support networks (such as volunteers in the surgery, or family members) were deemed especially valuable for those needing specific support to hear about, access and use the NHS App (such as those with language barriers), and a distinct barrier for those who did not have such assistance:

I would say it is a barrier, so when you’ve got patients that can’t speak that can’t read English it’s no good for them really…We still offer the services to them, [we] just ask them to have family or friends to help them with ordering their prescriptions online. (S11, Site 3, Admin Lead)

### Domain 5: the organisation

In the context of increasing demand pressures within practices during and following the pandemic, organisational capacity from general practices to innovate varied between case sites and was not always at the forefront of their efforts (see Table 1 for case study site characteristics and digital maturity rating). In one of the large practices already championing active engagement in digital innovations before the pandemic (site 1), there appeared to be more organic and unified processes for integration of new ways of working including dedicated practice staff with experience introducing new technologies:

… because of our involvement in that [working with industry] and other tools when Covid hit we were in quite a good position to be able to manage that …our patients were used to us communicating with them in that way [SMS messaging] we also I think because of my University background…where everything was very digital… I’d already been working over the previous year to try and get…people set up with things like VPN’s with remote access…And so actually and using more digital tools. (S1, Site 1, Practice Manager)

In other practices there was apprehension from practice managers that the introduction of digital innovations would conflict with a more ‘traditional’ organisational culture prioritising patients telephoning or attending face-to-face (site 2 and site 5):

…I know some practices have tried to introduce, not a barrier as such, but they have tried to really direct people sort of to, to remove the telephone option to some degree…I think because of our traditional approach, that would be something where I definitely will get resistance from the GPs…I don’t think they would want that as, as part of their practice. (S5, Site 2, Practice Manager)

There were also fears expressed by some staff around a lack of capacity to take in, organise and act on potential extra engagement or data as a result of digital innovations and augmented access to test results and health records. Organisational capacity and willingness to innovate, including leadership focus on innovation, led to varying levels of readiness for the NHS App and impacted on NHS App use and promotion within practices. Yet, when the app (or its add-on services) offered clear value by resolving or improving workflow, such as patients sending in photos in a secure and confidential way, it enabled organisational willingness to promote the use of the app even in the context of more ‘traditional’ organisational cultures (site 2):

so particularly sort of dermatology type things, we’ll encourage them [patients] to send photos and that’s, that’s where we’ve tried to use the [NHS] App, because that’s a secure way and the patient has had to prove who they are to gain access to the app etc. and so that’s the one we favour. (S6, Site 2, Practice Manager)

#### Nature of adoption and/or funding decisions

The introduction of the NHS App appeared to generate tension, confusion or conflict in practices already using products (apps or patient portals) with similar or additional (e.g. messaging or video consultations early on) functionality, with little support over how to navigate the digital landscape or reach a decision to switch to the NHS App. Integration of the COVID pass became a deciding factor as it increased awareness of the NHS App for patients and staff, yet some NHS App functions were still not promoted by practices with a history of using commercial apps or portals.

While the NHS App, in its basic form, does not require funding to use it, some add-on features, (which could potentially add unique or more valuable functions for the practice) do require funding. Yet, there was often a lack of dedicated income stream for practices, and so more innovative add-on features were seen as “out of reach”, even for practices that were more digitally focused. As a result, there were tensions between the value of the app set against funding, as well as infrastructure, organisational capacity, and staff roles.

### Domain 6: the wider context

The NHS App follows a long history of (more or less) successful initiatives to digitise care in the NHS. This includes attempts at a policy level to provide all patients with access to their own health records, which has been one of the most contested features at policy level [25].

I think this the, the patient, patient access programme has been a very challenging piece of work and, and it has yeah it’s become, become sort of fairly fraught…a lot of tension of kind of ways to make it happen but also to consider, consider all of the eventualities to consider safety. (SH15, stakeholder from professional body)

Controversy around this feature continued as the popularity of the NHS App was growing, with policymakers and professional bodies engaging in lengthy and difficult negotiations on how appropriate access would be enabled and how safeguarding issues would be avoided:

They [practices] think it’s massive safeguarding issues… they should be enabling patients to access their full records but the majority have not done that. This was in the GP contract way before the pandemic… policy wise…we’re leaving the burden of you [practices] having to do that by us doing it, they’re saying… [it] will increase burden because people will start seeing things that they didn’t see before and phoning up… and GP’s feeling like the clinical software to redact isn’t strong enough. (SH7, Development Team 2, Communications and Stakeholder Engagement)

National mass roll-out of access to prospective records was stalled and replaced by a less centralised approach with practices required to switch on access locally (including as part of new contractual requirements). Yet professional bodies continued to express concern and practice managers and professional bodies highlighted issues such as the range of considerations required to provide access while also protecting information that was not originally intended for sharing with patients:

We did have concerns about how well the programme had been communicated and practices, had some of them given enough time to prepare…and…checking the records for vulnerable patients who might need to have that…you can’t just drop a kind of a new piece of technology in the laps of extraordinary busy practices and expect them to be able to pick it up and make the best use of it… there’s a real need for the change management support … to see technology adopted but also… make sure that that it provides the outcomes and the benefits that it’s designed to. (SH15, stakeholder from professional body)

The idea of ‘cross-selling’ (as mentioned by one of the interviewees) also surfaced as one of the principles applied in the context of roll-out meaning that users registering on the NHS App for one purpose were then expected to find value in other functionality provided, such as organ donation and online prescriptions. In this respect, the COVID pass was seen as a major draw by some interviewees:

And we were hearing at the start of our work that people wanted it to be in the Covid-19 App because, well we’re already using [it]…people still might say that that’s the best place but for the App it’s had a huge amount of unintended consequences in terms of like organ donation registrations have gone through the roof. (SH3, Development Team 1, User Researcher)

However, others described the integration of the COVID pass on to the NHS App as ‘manipulative’ and setting a ‘terrible resonance’ (Data privacy organisation Coordinator). Their concerns centred primarily around data privacy and security when presenting the COVID pass (and subsequently the NHS App) in settings outside healthcare:

*… rather than having a Covid passport App they kludged it onto the NHS App, clearly they wanna force more people or encourage more people to, depending on your perspective, to use the NHS App. That’s a long-term win for them… [but] you’re talking about handing a, or showing a, a logged in device that’s having access to at least part of your medical records to a third party. (SH6, Data privacy organisation, Coordinator)*

From the perspective of the patients interviewed, there appeared to be less concern around data privacy, security and advertising compared to commercial products, given the NHS branding.

Wider context influencing the roll-out of the app included efforts at inter-organisational networking to support learning. In some instances, clinical commissioning groups became invested in the NHS App which they saw as a “nation[al] asset” they did not need to procure, and provided dedicated support for roll-out, including GP staff training, discussion forums between practices, and as a point of contact for queries. At the early stages of implementation, some areas even became designated ‘beacon sites’ which received additional assistance such as extra support and coaching but only over a limited time period. In other areas, commissioners did not perceive they had a mandate for the NHS App and engaged less in supporting roll-out.

### Domain 7: adaptation and sustainability over time

Given the NHS App primarily provides a gateway to different services and platforms, there is significant scope to add or remove functionality so the app remains relevant over time. This has been demonstrated already with the integration of the COVID pass, and subsequently with the addition of popular commercial services (at least in some areas) such as those facilitating online consultations and access to personal health records. This brings an inherent risk, however, in that the NHS App may end up incorporating too many features, making it difficult to sustain a coherent identity.

There is significant policy interest in maintaining interest and regular use of the NHS App, increasing functionality for purposes such as ‘day to day health management’ and securing engagement from patients and practices alike, beyond the COVID pass. Improvements and adaptations are being made over time, such as with the symptom checker (e.g. relevance for dermatological questions and different skin colours), increased security features, direct booking of vaccine appointments, and the addition of notifications to the app, although not all additions were perceived as useful by patient users:

Sometimes I get an email or notification saying my app has updated, like blood tests…and I go to check and sometimes the, where it tells me it has updated, are not on there… it doesn’t clarify what it means...I have to guess. (P26, Site 5, 34, Male, Black British)

Issues with digital inclusion will have to be considered, especially around embedding different languages and increasing patient-facing terminology as the user base grows.

## Discussion

### Summary of findings

Using the NASSS framework, this article highlights the multiple layers of complexity manifesting when introducing a shifting technology into a challenging environment such as English general practice, during and after a period of considerable societal turbulence in the context of the COVID pandemic. A number of interacting influences shaped the trajectory of the NHS App and we reflect on these below.

Rollout of the NHS App reflects and illuminates the complexity and fragmentation of the NHS, for example, when patients questioned why their information on the app appeared so disjointed across primary and secondary care. The app appeared to be less able to support those accessing ongoing health care outside of general practice (for example, patients we spoke to who were living with Parkinson’s, HIV, and diabetes) to manage their condition(s), yet useful for those with conditions primarily managed in primary care (for access to prescription ordering, NHS number, GP related letters, records of GP consultations, test results). The app was also perceived by some staff and patients as being less useful for those without long-term conditions.

There was a level of health system literacy required to comprehend how to access, or request support to access, the app, and understand what the NHS app was, and what functions it encompassed (in addition to being the COVID Pass). To a certain extent, adoption relied on general practice and its staff, who assumed responsibility in supporting patients to access the app but also in safeguarding patients, by restricting access to health records where needed. The app also required integration and access to a number of background systems and IT infrastructures, which meant that not all features were available by default by all users (e.g. own health records). Further, practices made decisions about what features would be available, e.g. appointment booking, so this function, which was desired by many patients, was not available for all. The app design offered a transactional, mechanistic approach to functionality such as appointment booking, which did not translate well into complex practice processes that often relied on relationships with patients. This was also the case for patient access to own records, which were controlled by practices, and for which procedures for access were variable and not understood by all staff, or where staff were concerned about GDPR (and requirements for redaction).

While policy-makers saw the app as a “front door” to the NHS for patients, the purpose and utility of the app, or how this shifted over time, was perceived as not being satisfactorily communicated or fully co-designed in collaboration with practices. However, the COVID pass made the presence and value of the app to practices and the wider public more prominent and coincided with a drive-in user base [16]. This was despite development teams having carried out substantive user research, including on accessibility, that remained focused, however, on use by patients rather than the complex work of other implicated groups such as healthcare staff. There was also heterogeneous support provided to, and by, CCGs, which created inconsistent experiences and approaches to roll-out.

For patients, features such as ordering prescriptions online held a strong value proposition. Security features, e.g. fingerprint scanning and face recognition, were appreciated, especially by those with health diagnoses they did not want to be shared. Accessing their own health records (including proxy access) engendered an increased sense of control over health for many patients, as well as enabling a sense of ensuring accountability of the health service. However, there was complexity and variability in the value proposition of the NHS App, as different features were added, removed or became more or less relevant over time (e.g. COVID pass). NHS App use has been fundamentally influenced by external events such as the COVID-19 pandemic, which had a clear impact on uptake, with evidence of increases in app adoption at the onset of the first national lockdown and a fourfold increase in downloads following the introduction of the COVID-19 Pass [16]. However, events of such magnitude are rare, which means part of the learning around how the app was received may not be directly transferable, yet most of the lessons still apply beyond the pandemic context.

Despite interest in patient access to their own records through the NHS App, finding a balance between providing access and safeguarding, without imposing additional workload on practices has been challenging. Related issues have been highlighted in previous research. For example, Louch et al. discuss how clinical and non-clinical staff in primary care supported patient access but also voiced concerns about the type of historical information that would become available and how this might be interpreted or used by those accessing this information [26]. Along similar lines, Nøst et al. found healthcare professionals feared that increased access could increase workload due to additional need for patient follow-up [27]. From a patient perspective, McMillan et al. report the need to better promote availability of access, as well as introduce joined-up records with community and secondary care [28]. However, complexity is exhibited when providing access to features such as health records, which does not automatically lead to patient ‘empowerment’ (as shown elsewhere [29]), especially when considerable work is required from patients to access such features.

From an organisational perspective, several studies have identified barriers and facilitators related to the implementation of patient portals [30, 31]. Our study further exemplifies how multiple interacting influences co-shape the trajectory of a complex technology with evolving components reaching various parts of the healthcare service, particularly so in a crisis context. The involvement of health professionals in the design and implementation of patient portals plays an important role in mediating their experiences [32]. GPs have previously expressed that they want to feel more supported with digital change (in procurement or implementation) and that innovation-focused practice leadership (as found in this study), and support from the PCN or commissioning body around implementation has enhanced implementation [33]. Finding an optimal balance between top-down and bottom-up implementation remains challenging but is necessary to enable alignment with local care processes and priorities [34].

The motivation at the organisational level to put in place the processes, staff roles and capacity required for the change effort changed in the context of increasing demand and work pressures during and following the pandemic. The availability, interest and capacity of healthcare staff to support adoption also varied in the context of what was perceived as top-down implementation, and in terms of the cultural context of practices e.g. apprehension from practices with a more ‘traditional’ organisational culture, as opposed to those practices who were more innovation-focused and digitally strategic (according to Greenhalgh’s digital maturity scale [18]).

There were considerable influences from the wider context on NHS App roll-out, which shifted from a ‘remote-by-default’ orientation to an emphasis on in-person care, as well as controversies related to specific features (such as centralised patient access to own records). Where inter-organisational working, e.g. between practices and commissioning groups occurred, the additional support encouraged rollout in practices. In this landscape, the NHS App offered a means of extending previous efforts to introduce online access to services in the UK [35, 36]. Improvements and adaptations informed by user research were being made to the app throughout data collection, although there were challenges with digital inclusion as well as maintaining user interest in an offer that would remain relevant over time.

Issues of health equity have also been debated in previous literature which explains some of our findings, that disproportionate emphasis is placed on individual responsibility to access and use patient portals, without sufficient acknowledgement of system-level barriers and support for underserved populations [37]. This transcended through patient participant’s experiences of requiring support to log in to and access the NHS App and its various features. Further, those from more affluent, white and middle-aged backgrounds with high health literacy appear to be more likely to use digital tools such as patient portals [37]. In our wider evaluation of the NHS App, there were higher rates of app uptake among practices in less deprived areas and with patients who were generally younger, white and with lower healthcare needs [16]. Such trends of inequalities implicate a real risk of a new digital Inverse Care Law whereby attempts at digital transformation directly or indirectly compromise health equity [38, 39]. With primary care being the access point for many to the health service, there is a pronounced need to consider the complex factors influencing adoption and roll-out and to ensure that the adoption of digital health tools do not create or exacerbate inequities in health service access or use.

### Strengths and limitations

We were able to recruit a diverse sample of research participants who varied in terms of age, ethnicity, health conditions, location, health and digital literacy. Study sites also covered a wide range of characteristics and patterns of technology engagement across the country. By using different qualitative research methods over time, we were able to generate a rich understanding of the complexity underpinning the rollout of the NHS App and ensure that we were recruiting diverse perspectives. We found that most participants where current users of the app, and many participants would refer to their app during interviews and focus groups. Further, proxy users that we spoke to provided a dual perspective as someone who had access to the NHS App for themselves, and also on behalf of a family member.

Yet we also faced challenges during the course of the study in the pandemic and post-pandemic context which may limit some of the transferability and reach of the findings. Further, the NHS App interface and functionality changed at pace which meant it was difficult to pin down what version of the app patients were referring to in remote interviews. However, capturing this experience of patients also gave more emphasis to the complexity of this app, the app’s integration with practices, the presentation to patients, and the lack of comprehension, at times, about what the app’s functions and purpose were.

There was limited engagement with those who face language barriers (with the app only available in English) and those who face wider disadvantages across various characteristics that influence the extent to which they would engage with digital options. Future research should focus on the specific needs of disadvantaged groups in relation to access to primary care through the NHS App, as well as on the specific needs of GP providers with different levels of digital readiness when it comes to supporting digital options.

## Conclusions

In this qualitative study, we aimed to unpack the complexity underpinning the implementation, use and roll-out of the NHS App, learning from the experiences of patients (users and non-users), healthcare staff and other relevant stakeholders. We found that patients had diverse (positive and negative) user experiences as the app evolved, with some of its features described as more useful (e.g. prescription ordering or access to records). Yet the app was primarily a gateway to general practice systems and infrastructures, therefore, not all features were available by default and consistently to all users, with information often appearing fragmented or presented in a system-facing way (e.g. coded). Using the app as a “front door” to the health service also assumed a certain level of system literacy which was not always realised. For practices, although the app did not initially appear to have a clear value proposition in terms of supporting their work or engagement with patients, the scope for potential future development as core NHS infrastructure made it appealing. As the NHS App remains a complex intervention in a complex landscape, it is clear ongoing work is needed to ensure the app can sustain its potential and will continue to meet patient, service and policy needs.

## Supplementary Information

Supplementary Material 1: Supplementary information 1: Images of the app from patient interviews.

Supplementary Material 2. Consolidated criteria for reporting qualitative studies (COREQ): 32 item checklist.

Supplementary Material 3. NHS Staff interview topic guide.

Supplementary Material 4. Patient and Carer Focus Group topic guide.

Supplementary Material 5. Patient interview topic guide.

Supplementary Material 6. Stakeholder interview topic guide.

## Data Availability

No datasets were generated or analysed during the current study.

## Abbreviations

CCG: Clinical Commissioning Group
CEO: Chief executive officer
COVID-19: Coronavirus disease 2019
GDPR: General Data Protection Regulation
GP: General Practitioner
HIV: Human immunodeficiency virus
ICS: Integrated care systems
IMD: Index of Multiple Deprivation
LCRNs: Local Clinical Research Networks
NASSS Framework: Non-Adoption, Abandonment, Scale-up, Spread and Sustainability framework
NHS: National Health Service
PPI: Patient and Public Involvement
