# Supplementary material for: Qualitative evaluation of the implementation and national roll-out of the NHS App in England
Source: BMC Med. 2025 Jan 21;23:20. doi: 10.1186/s12916-024-03842-w (PMC11752663; doi:10.1186/s12916-024-03842-w)
Supplement: Supplementary file 1 — Supplementary Material 1: Supplementary information 1: Images of the app from patient interviews. [file 12916_2024_3842_MOESM1_ESM.docx]

# Supplementary information 1: Images of the app from patient interviews


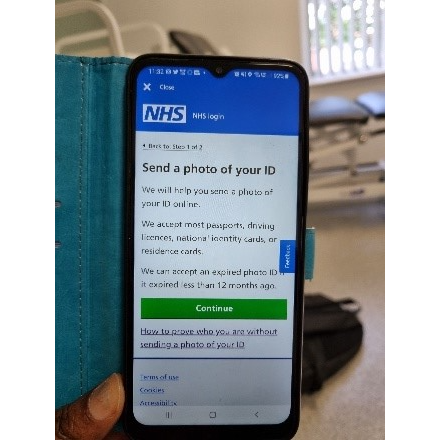


Image 1: Site 4, P25 – Patient proof of identification required to register with the app.


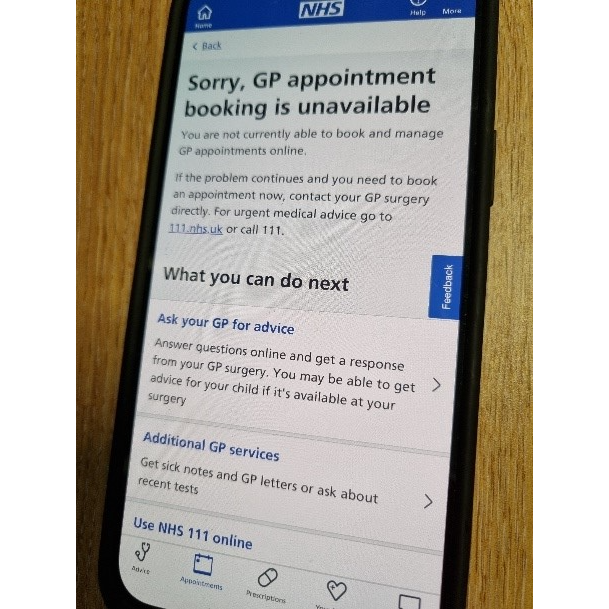


Image 2: Site 4, P23 - Appointment booking unavailable


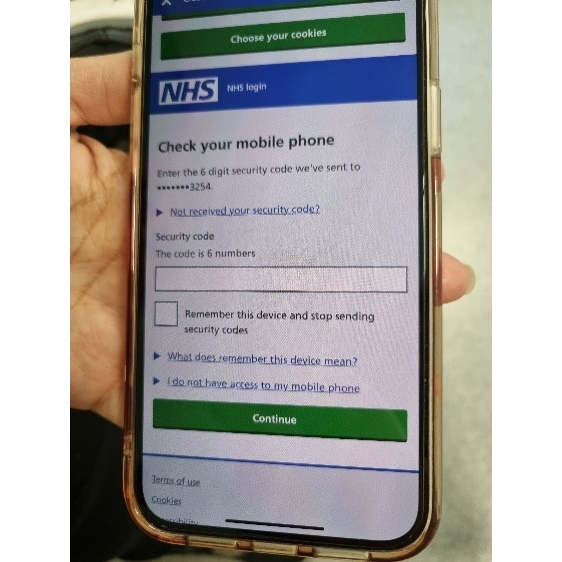


Image 3: Site 4, P24 - Security features


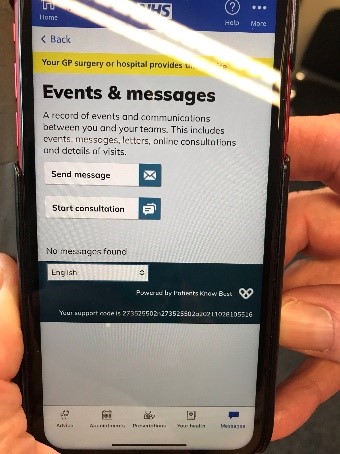


Image 4: Site 2, P3 - Additional services through industry partners (messages to secondary care providers)


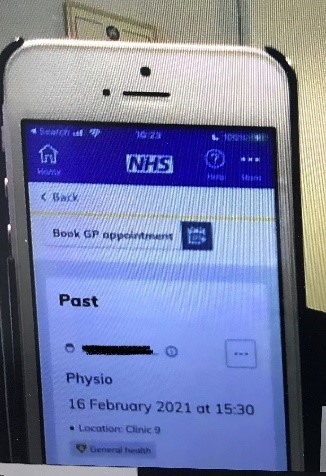


Image 5: Site 2, P4 - Past appointment viewing
