# Supplementary material for: Qualitative evaluation of the implementation and national roll-out of the NHS App in England
Source: BMC Med. 2025 Jan 21;23:20. doi: 10.1186/s12916-024-03842-w (PMC11752663; doi:10.1186/s12916-024-03842-w)
Supplement: Supplementary file 2 — Supplementary Material 2. Consolidated criteria for reporting qualitative studies (COREQ): 32 item checklist. [file 12916_2024_3842_MOESM2_ESM.docx]

**Consolidated criteria for reporting qualitative studies (COREQ): 32 item checklist**

| No. | Item | Guide Q’s/description | Author response | Location in paper |
| --- | --- | --- | --- | --- |
| Domain 1: Research team and reflexivity | | | | |
| Personal characteristics | | | | |
| 1 | Interviewer/facilitator | Which author/s conducted the interview or focus group? | Lead authors CR and CP | See manuscript title page, Page 1 |
| 2 | Credentials | What were the researcher’s credentials? E.g. PhD, MD | Both researchers hold a PhD | See manuscript title page, Page 1 |
| 3 | Occupation | What was their occupation at the time of the study? | Health Services Researcher and Associate Professor |  |
| 4 | Gender | Was the researcher male or female? | Female |  |
| 5 | Experience and training | What experience or training did the researcher have? | Many years of qualitative research experience and teaching |  |
| Relationship with participants | | | | |
| 6 | Relationship established | Was a relationship established prior to study commencement? | No |  |
| 7 | Participant knowledge of the interviewer | What did the participants know about the researcher? e.g. personal goals, reasons for doing the | The participants knew what the roles and experience of the researchers were and the reasons for doing this research |  |
| 8 | Interviewer characteristics | What characteristics were reported about the interviewer/ facilitator? e.g. Bias, assumptions, reasons and interests in the research topic | Participants were told that we were undertaking an independent evaluation of the NHS App roll out, using qualitative methods, and that we were interested in understanding the range of experiences of perceptions about the app, and that this knowledge could also contribute to understanding about the roll out of some other apps in primary care |  |
| Domain 2: study design | | | | |
| Theoretical framework | | | | |
| 9 | Methodological orientation and Theory | What methodological orientation was stated to underpin the study? research e.g. grounded theory, discourse analysis, ethnography, phenomenology, content analysis | The NASSS framework | Page 10 – Methods – data analysis, theoretical framework |
| Participant selection | | | | |
| 10 | Sampling | How were participants selected? e.g. purposive, convenience, consecutive, snowball | In paper | See page 7, Methods – data collection - Participant recruitment and sampling |
| 11 | Method of approach | How were participants approached? e.g. face-to-face, telephone, mail, email | In paper | See page 7, Methods – data collection - Participant recruitment and sampling |
| 12 | Sample size | How many participants were in the study? | In paper | See page 6, Methods – Data collection |
| 13 | Non-participation Setting | How many people refused to participate or dropped out? Reasons? | No one who initially showed interested in taking part dropped out or refused to participate. Potential participants responded to open invitations and so we only knew about those who had shown an interest in taking part. |  |
| 14 | Setting of data collection | Where was the data collected? e.g. home, clinic, workplace | Some data was collected in a clinic, and some were online (either at home for patients or in the workplace for clinicians, stakeholders and patients), see in text description as well. | See page 6 and 7, Methods – Data collection |
| 15 | Presence of non-participants | Was anyone else present besides the participants and researchers? | No |  |
| 16 | Description of sample | What are the important characteristics of the sample? e.g. demographic data, date | See Table 2 for participant characteristics | See page 7 and 8, Methods – data collection - Participant recruitment and sampling |
| Data collection | | | | |
| 17 | Interview guide | Were questions, prompts, guides provided by the authors? Was it pilot tested? | We used a specific topic guide for each interview / focus group type. It was discussed and examined with our study PPI group before being used. | Attached as supplementary files |
| 18 | Repeat interviews | Were repeat interviews carried out? If yes, how many? | No |  |
| 19 | Audio/visual recording | Did the research use audio or visual recording to collect the data? | All interviews and focus groups were digitally recorded and transcribed verbatim for analysis. Think aloud interviews were video recorded. | See page 6 and 7, Methods – Data collection |
| 20 | Field notes | Were field notes made during and/or after the interview or focus group? | Field notes were taken for observations and interviews and focus groups | See page 6 and 7, Methods – Data collection |
| 21 | Duration | What was the duration of the interviews or focus group? | Interviews and focus groups lasted between 16-72 minutes (average=41 minutes) and 59-87 minutes (average=73 minutes) respectively | See page 6 and 7, Methods – Data collection |
| 22 | Data saturation | Was data saturation discussed? | Yes, in paper | Page 10 and 11– Methods – data analysis, analysis |
| 23 | Transcripts returned | Were transcripts returned to participants for comment and/or correction? | No, but anonymised transcripts were discussed with our study PPI group |  |
| Domain 3: analysis and findings | | | | |
| Data analysis | | | | |
| 24 | Number of data coders | How many data coders coded the data? | 2 authors coded the data (CR and CP) |  |
| 25 | Description of the coding tree | Did authors provide a description of the coding tree? | See the NASSS Framework domains | Page 10 – Methods – data analysis, theoretical framework |
| 26 | Derivation of themes | Were themes identified in advance or derived from the data? | Braun and Clarke’s six-step framework guided our thematic analysis, which began with data familiarisation, generation and iterative refinement of initial ideas and codes from transcripts and fieldnotes (22). This was followed by inductive and deductive development of broader themes within and across NASSS domains, including comparing and contrasting similarities, differences, and connections in the data (especially across sites). Combining an inductive and deductive approach allowed us to pay attention to emergent as well as anticipated themes and to identify cross-cutting areas of complexity. We used NVivo 12 for data management. | Page 10 and 11– Methods – data analysis, analysis |
| 27 | Software | What software, if applicable, was used to manage the data? | We used NVivo 12 for data management. | Page 11– Methods – data analysis, analysis |
| 28 | Participant checking | Did participants provide feedback on the findings? | Our Patient and Public Involvement (PPI) group was involved in the process of data analysis and sense-making through six workshops held at project initiation and set-up, through recruitment, and as data emerged. Further, weekly team meetings with the PPI lead facilitated continued engagement throughout the study. | Page 11– Methods – Patient and Public Involvement contribution |
| Reporting | | | | |
| 29 | Quotations presented | Were participant quotations presented to illustrate the themes / findings? Was each quotation identified? e.g. participant number | Yes | See pages 11- 19, Results |
| 30 | Data and findings consistent | Was there consistency between the data presented and the findings? | Yes | See pages 11- 19, Results, and page 20 – 22, Discussion |
| 31 | Clarity of major themes | Were major themes clearly presented in the findings? | Yes | See pages 11- 19, Results |
| 32 | Clarity of minor themes | Is there a description of diverse cases or discussion of minor themes? | Yes, detailed descriptions of more distinct and more minor themes throughout |  |
