# Supplementary material for: Qualitative evaluation of the implementation and national roll-out of the NHS App in England
Source: BMC Med. 2025 Jan 21;23:20. doi: 10.1186/s12916-024-03842-w (PMC11752663; doi:10.1186/s12916-024-03842-w)
Supplement: Supplementary file 3 — Supplementary Material 3. NHS Staff interview topic guide. [file 12916_2024_3842_MOESM3_ESM.docx]

**NHS Staff interview topic guide**

**Study title: Evaluating the national rollout of the NHS App in England**

**Introduction to the study**

Thank you for agreeing to take part in the study - Evaluating the national rollout of the NHS App in England.

Do you have any questions about the study?

**Consent**

Take verbal consent

**Topic guide**

Tell me about your service/role/wider team. Have you been working long in this profession and in the current practice?

What do you know about the NHS App?

How is your practice integrated to the different functions the app provides, e.g. booking appointments? Is the practice using any other means to provide patients with digital access to services?

Who is responsible for integrating the app with back-end workflows and how do they keep things going when/if problems arise?

What support/training have you/others had to use/support use of/implement the app?

Have you had any discussions with patients about the NHS App? Have patients been seeking support to use it, or has anyone had questions about, e.g. their medical details on the app?

Could you provide specific examples/stories where you have found the app helpful (e.g. in the context of a clinical consultation) or when use of the app created difficulties (e.g. not being able to set up appointment booking)?

Has the Covid-19 crisis had an impact on the way the app was deployed by your practice?

How do you think the NHS App might influence (support or hinder) patient access to health services?

Is there anything you would change about the app and the way it integrates with the service currently? Would you change anything about the way the app has been introduced to the public?

**Demographics of participant**

In order to ensure we are speaking to a wide range of people, we just need to ask a few basic demographic questions about you:

- How old are you?
- Which gender do you most identify with?
- How would you describe your ethnic origin?

Thanks and seek suggestions for other interviewees
