# Supplementary material for: Qualitative evaluation of the implementation and national roll-out of the NHS App in England
Source: BMC Med. 2025 Jan 21;23:20. doi: 10.1186/s12916-024-03842-w (PMC11752663; doi:10.1186/s12916-024-03842-w)
Supplement: Supplementary file 4 — Supplementary Material 4. Patient and Carer Focus Group topic guide. [file 12916_2024_3842_MOESM4_ESM.docx]

**Patient and Carer Focus Group topic guide**

**Study title: Evaluating the national rollout of the NHS App in England**

Thank you for agreeing to take part in the study - Evaluating the national rollout of the NHS App in England

Firstly, we would like to introduce ourselves. We work in the Nuffield Department of Primary Care Health Sciences, at the University of Oxford, my name is Claire Reidy, Health Services Researcher and Chrysanthi Papoutsi, Senior Researcher.

**Introduction to the study**

**What we’re doing**

This research is looking at what different groups think about the NHS App, how they use it, whether they find the app helpful, and whether it changes how people use the NHS.

We are talking to patients, carers, clinical and non-clinical staff, those who commission health services, technology developers and policy-makers to explore the use and roll out of the NHS App.

**Independent evaluation**

This is an independent, and impartial evaluation of the NHS App, so what you say will be de-identified and kept confidential, we have no involvement in the NHS App so we hope that you can be honest with us about your experience and thoughts about the NHS App.

We will analyse the data we collect from our discussions with people and will feed back key findings to teams developing the NHS App, but we will do this without disclosing any data that may identify participants.

**Safety/confidentiality/withdraw**

We will record this focus group and have it transcribed. Any identifiable information will be removed from the transcript so you will not be able to be identified from the transcript. All data collected will be stored securely and password protected on university machines.

Before we start we would like to reassure you that you can withdraw from this Focus Group, and the study at any time without providing a reason and without prejudice; and your usual care will not be affected.

Should you feel upset, we can move onto a different question, take a break in the Focus Group, step out of the room, or terminate the Focus Group altogether. You can do this, at any time.

This decision will not affect any services you or your relatives receive and will not affect your legal rights.

Do you have any questions about the study?

**Consent**

So, in order to take verbal consent I will read some questions out and can you please each let me know whether you are happy to continue to take part? I will also send you a copy of the consent form by email.

**But before we get started, could we go around and share our names?**

**Ground rules**

So, in terms of ground rules for the study, I will put forward some questions and hopefully we can discuss these as a group. If someone is talking, please raise a virtual hand to show that you would like to speak next.

Ideally we can keep to a live discussion, but if there is not time to make a point please do add your point in the chat and we’ll include this in the discussion too.

Please also respect each other’s opinions, there are no right or wrong answers and we would really appreciate hearing each of your thoughts and experiences about using the NHS App.

**Topic guide:**

**Introductory (10-15 minutes)**

1. **So you have all heard about the NHS App, going back to when you first start using the app – do you remember why you first started using the app?**
2. **Does anyone have any particular difficult experiences of using the app, or times where the app has been particularly helpful?**
3. **Do you have any concerns about the app?**
   - Have you been using the NHS App for a long time?
   - did you use it before covid-19?
4. **How easy it was to download and register with the app?**
   - How did you manage the log-in process? Did you face any difficulties or did you need support to start using the app?
5. **Did anyone support you to access and use the app**?
   1. Have you supported anyone else to use the app?

**Core part:**

Do you find you need to use the app often?

What do you use it for?

- 1. What you find most helpful and why? What you find least helpful?
  2. Do you use it to help manage any health conditions you have? And have you had any problems using it this way? Examples – tell us about a time when you used the app, how it helped, how it didn’t help…
  3. Is there anything you would like to access on it but can’t?
     1. Or could before but cannot now?

Are there things you find easier doing on the app compared to how you did them previously (e.g. prescriptions)

- - 1. Can you give me any examples?

Has anyone used the NHS App to (suggestions/reminder for what is on the app);

- order repeat prescriptions
  1. choose a pharmacy for your prescriptions to be sent to
- see your available medicines
- consult a GP or health professional through an online form and get a reply
- get your NHS COVID Pass
- view your NHS number (or find out what it is!)
- search for, book or cancel appointments at your GP surgery
- see details of your upcoming and past appointments
- view your health record
  1. see information like your allergies
  2. your current and past medicines.
  3. see your like test results
  4. See details of your consultations
- message your GP surgery or a health professional online
- get advice about coronavirus
- get health advice
- look up referrals
- view your hospital and other healthcare appointments
- view letters send from the hospital or other clinic outside of the GP surgery
- register your organ donation decision
- find out how the NHS uses your data - choose if data from your health records is shared for research and planning
- access health services on behalf of someone you care for –
  1. if so, how?
- view any links your doctor or health professional has shared with you
- view and manage care plans – additional services?

**How has the NHS App influenced how you access your GP surgery, or other health services?**

- - **What did you do before?**
- Has the app enabled you to access health services in a different way to before? e.g. speaking to your GP more easily/access health records/results that you did not have access to before (can you give some examples?)

Tech use - **Do you use any other health apps/websites apps/websites in the context of managing your health?**

- Have you used any other ways to book appointments with your GP practice or view your records/test results online?
- Pros and cons of different options, examples

**Is there anything you would change about the way the app works currently?**

- Would you suggest any additional functions or improvements?

**Vaccine passport**

Have you heard about recent developments of the NHS App being used as a vaccine passport?

- - Have you accessed your vaccine passport/COVID pass on your app?

**What do you think about the NHS App being used as a vaccine passport?**

- - (Is it about technical issues, or something about the app?)
  - What are the pros of using the app as a vaccine passport vs paper based vaccine passport
  - What are the cons of using the NHS App as a vaccine passport vs paper based vaccine passport

Do you feel there are any groups this would be more beneficial for? Challenges? Benefits?

**Would you use the app as a vaccine passport?**

- **Has it made a difference as to how you use the NHS App?**
- Is there anything you would change about the way the NHS App is being used as a vaccine passport?
- **What do you think about… the app was also used as a national vaccine passport? (In order to access live music venues, restaurants, pubs, etc.)**

(For non-users)

What stopped you from accessing and using the app?

How do you normally book appointments with your GP practice and access your medical details?

What do you think about the way this works at the moment (e.g. is it easy to access your practice)?

**Closing**

We have now covered all of the questions we were hoping to discuss with you all. Is there anything else that anybody would like to add? Thank you all so much for taking part. Your contribution has been so valuable and so interesting, and helpful.

We will continue to undertake more focus groups and once these have all been transcribed we will analyse the data that we have. We will write a report based on the information that we have and will write an academic paper with this data. We will feedback the information that we find to the teams that have developed the NHS App in order to improve the experience of accessing and using the app and the roll out of the app nationally. We also hope to share the information that we find in blogs and with different patient groups, and healthcare professionals.

If you would like to receive a summary of the results please can you let me know and I can send you a summary at the end of the study in September 2022.

I will send you your voucher in an email over the next week.
