# Supplementary material for: Qualitative evaluation of the implementation and national roll-out of the NHS App in England
Source: BMC Med. 2025 Jan 21;23:20. doi: 10.1186/s12916-024-03842-w (PMC11752663; doi:10.1186/s12916-024-03842-w)
Supplement: Supplementary file 5 — Supplementary Material 5. Patient interview topic guide. [file 12916_2024_3842_MOESM5_ESM.docx]

**Patient and Carer interview topic guide**

**Study title: Evaluating the national rollout of the NHS App in England**

**Introduction to the study**

Thank you for agreeing to take part in the study - Evaluating the national rollout of the NHS App in England.

Do you have any questions about the study?

**Consent**

Take verbal consent

**Demographics of participant**

In order to ensure we are speaking to a wide range of people, we just need to ask a few basic demographic questions about you. You are not obliged to answer any of the following questions but the more of the questions you answer the more we can understand who we are speaking to.

- How old are you?
- Which gender do you most identify?
- How would you describe your ethnic origin?
- How would you describe your experience with technology use?

**Topic guide:**

Do you find you need to access NHS services often in the context of managing your health?

Would you like to tell me a bit more about the things you need to manage related to your health, e.g. long-term physical or mental health conditions, or anything else you’d like to share?

Does anyone help you to manage your health (e.g. family, friends, neighbours, professional carers or support workers)?

Have you heard about the NHS App, and if so, do you remember how you found out about it?

How did you access to the app? How did you manage the log-in process? Did you face any difficulties or did you need support to start using the app?

How have you used the NHS App so far? Approx. how often would you say you use it/access information on the app?

(if relevant) Do you have caring responsibilities of others?

Have you used the app to support the care of people you are looking after? If so, how?

Which of the features do you find most helpful? Why?

Which of the features do you find less helpful? Why?

How has the NHS App influenced (supported or hindered) the way you access health services?

Have you been able to do things that you may not have been able to do otherwise? Can you give me some examples?

Have you tried to do things that you found were not feasible? Can you give me some examples?

How have you used the app in the context of receiving care during the Covid-19 crisis?

Do you use any other health apps/websites? Have you used any other ways to book appointments with your GP practice or view your records/test results online?

Is there anything you would change about the way the app works currently? Would you suggest any additional functions or improvements?

(For non-users)

What stopped you from accessing and using the app?

How do you normally book appointments with your GP practice and access your medical details?

What do you think about the way this works at the moment (e.g. is it easy to access your practice)?

Do you use any other apps/websites in the context of managing your health?
