# Supplementary material for: Qualitative evaluation of the implementation and national roll-out of the NHS App in England
Source: BMC Med. 2025 Jan 21;23:20. doi: 10.1186/s12916-024-03842-w (PMC11752663; doi:10.1186/s12916-024-03842-w)
Supplement: Supplementary file 6 — Supplementary Material 6. Stakeholder interview topic guide. [file 12916_2024_3842_MOESM6_ESM.docx]

**Stakeholder interview topic guide**

**Study title: Evaluating the national rollout of the NHS App in England**

**Introduction to the study**

Thank you for agreeing to take part in the study - Evaluating the national rollout of the NHS App in England.

Do you have any questions about the study?

**Consent**

Take verbal consent

**Topic guide**

Tell me about your role/wider team/organisation. Have you been working long in this role and in this organisation?

Can you tell me how you have been involved with the development/deployment/roll-out of the NHS App?

What were the key challenges you identified in development/deployment/roll-out?

How do you think the app might influence patient access to and use of primary care?

What else do you think the app might help with?

What do you think about the way the NHS App has been implemented so far? Does the app do what it set out to do?

How did the Covid-19 crisis influence the way the app has been developed or deployed?

Where does the NHS App sit in relation to other patient access solutions or similar digital health tools?

How do you expect the app will develop in the future?

**Demographics of participant**

In order to ensure we are speaking to a wide range of people, we just need to ask a few basic demographic questions about you:

- How old are you?
- Which gender do you most identify?
- How would you describe your ethnic origin?
